# Supplementary material for: DMRforPairs: identifying Differentially Methylated Regions between unique samples using array based methylation profiles
Source: BMC Bioinformatics. 2014 May 15;15:141. doi: 10.1186/1471-2105-15-141 (PMC4046028; doi:10.1186/1471-2105-15-141)
Supplement: Additional file 2 — DMRforPairs output for the comparison of A431-MCF7 and NA17018-NA17105. Please start from the HTML files in each folder. Available via the BMC Bioinformatics website. [file 1471-2105-15-141-S2.zip › 1394847754114233_MOESM2_ESM/A431_MCF7/figures/1446.pdf]

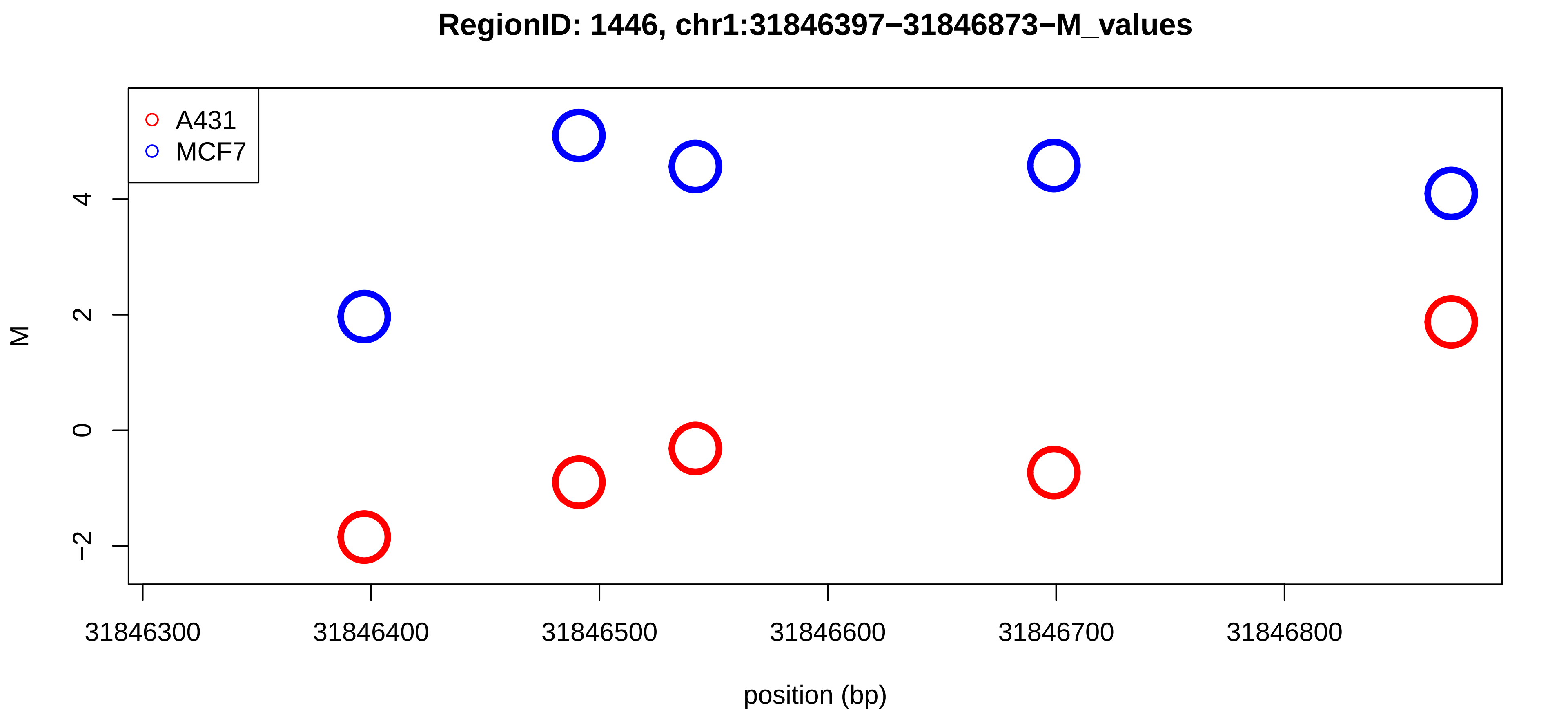

RegionID: 1446, chr1:31846397−31846873−M_values
A431
MCF7
M
position (bp)
31846300
31846400
31846500
31846600
31846700
31846800


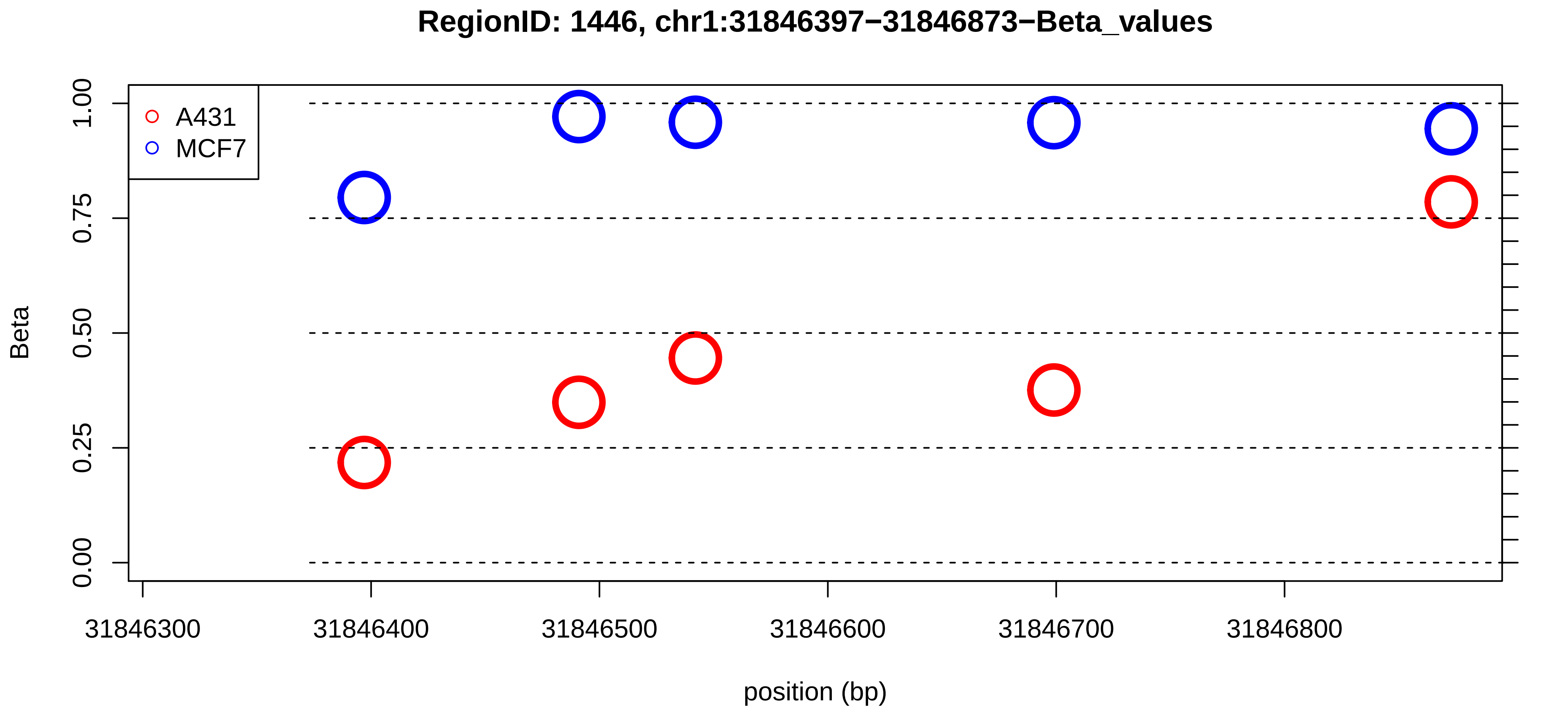

RegionID: 1446, chr1:31846397−31846873−Beta_values
A431
MCF7
Beta
position (bp)
0.00
0.25
0.50
0.75
1.00
31846300
31846400
31846500
31846600
31846700
31846800

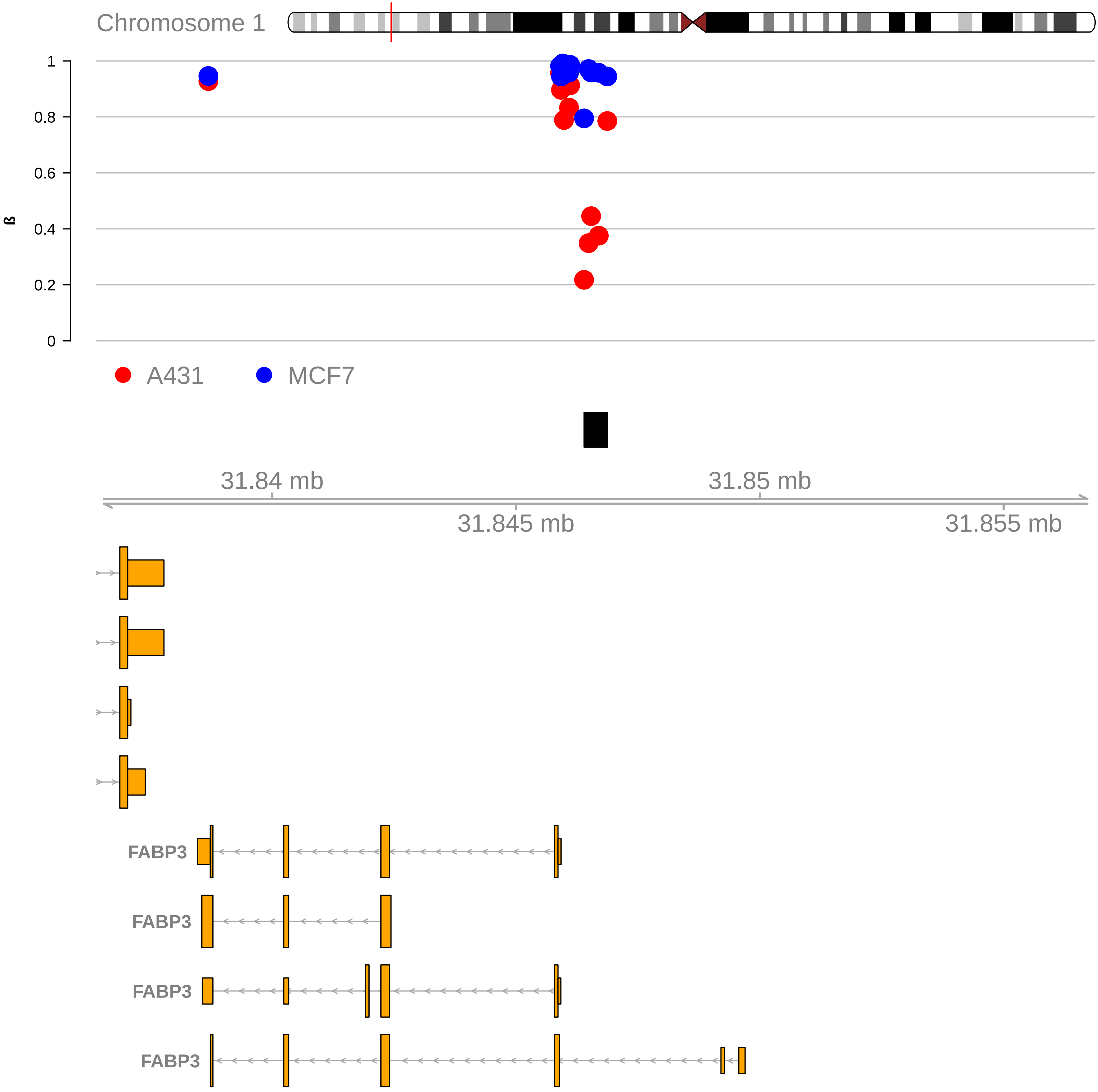

Chromosome 1
1
0.8
0.6
0.4
0.2
0
ß
A431
MCF7
31.84 mb
31.85 mb
31.845 mb
31.855 mb
FABP3
FABP3
FABP3
FABP3
